# Differences and similarities between SARS-CoV and SARS-CoV-2: spike receptor-binding domain recognition and host cell infection with support of cellular serine proteases

**DOI:** 10.1007/s15010-020-01486-5

**Published:** 2020-07-31

**Authors:** Giovanni A. Rossi, Oliviero Sacco, Enrica Mancino, Luca Cristiani, Fabio Midulla

**Affiliations:** 1 Department of Pediatrics, G. Gaslini University Hospital, Genoa, Italy; 2 Department of Maternal Science, Sapienza University of Rome, Rome, Italy

**Keywords:** Sars-CoV-2, Furin, Receptor binding domain, Sars-CoV, Coronavirus

## Abstract

Novel severe acute respiratory syndrome coronavirus-2 (SARS-CoV-2) became pandemic by the end of March 2020. In contrast to the 2002–2003 SARS-CoV outbreak, which had a higher pathogenicity and lead to higher mortality rates, SARSCoV-2 infection appears to be much more contagious. Moreover, many SARS-CoV-2 infected patients are reported to develop low-titer neutralizing antibody and usually suffer prolonged illness, suggesting a more effective SARS-CoV-2 immune surveillance evasion than SARS-CoV. This paper summarizes the current state of art about the differences and similarities between the pathogenesis of the two coronaviruses, focusing on receptor binding domain, host cell entry and protease activation. Such differences may provide insight into possible intervention strategies to fight the pandemic.

## Introduction

At the end of December 2019, Chinese public health officials announced to the World Health Organization (WHO) that a novel virus caused in Wuhan a disease with symptoms similar to pneumonia [1]. They recognized that the virus was from the coronavirus family and was formally named severe acute respiratory syndrome coronavirus-2 (SARS-CoV-2)**.** SARS-CoV-2 rapidly became pandemic by the end of March 2020, forcing much of the world to adopt lockdown strategies and putting health care systems under pressure while major concern about global health and economic stability arose. In contrast to the 2002–2003 SARS-CoV outbreak, which had a higher pathogenicity and lead to higher mortality rates, SARS-CoV-2 infection appears to be much more contagious, rapidly spreading to all continents. Compared to SARS-CoV, SARS-CoV-2 infection is characterized by a wider clinical spectrum, including asymptomatic infection, mild upper respiratory tract illness, severe viral pneumonia with respiratory failure and death [1, 2]. In contrast to SARS-CoV, many SARS-CoV-2-infected patients are reported to develop low-titer neutralizing antibody and usually suffer prolonged illness, suggesting a more effective SARS-CoV-2 immune surveillance evasion than SARS-CoV [3, 4]. Since the high transmission rate and viral immune escape may be involved in the SARS-CoV-2 widespread, both potentially representing a target for interventional strategies, it is of utmost importance to elucidate the molecular mechanisms which are involved in these atypical pathogenetic features.

## Coronaviruses structure and replication

**Fig. 1:**
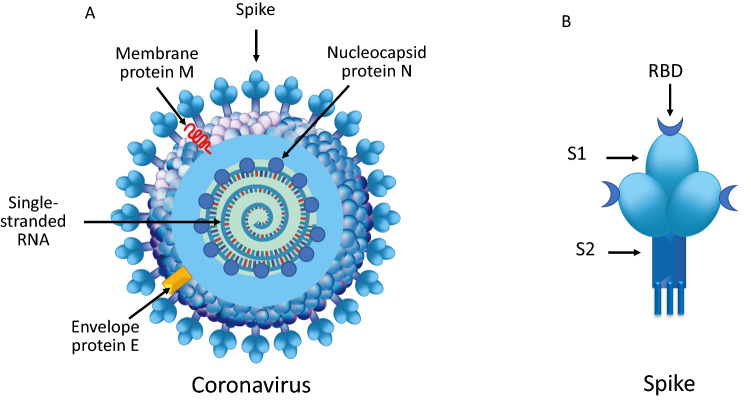
**a** Coronaviruses structures. The membrane (M), envelope (E) and spike (S) structural proteins are anchored to the viral envelope which contains the ribonucleoprotein core, i.e., the nucleocapsid protein (N) which acts as a scaffold surrounding the single-stranded RNA. **b** The surface spike is composed by the S1 subunit, which harbors the receptor binding domain (RBD), and by the S2 subunit, the stem which anchors the spike to the viral envelope and, following protease activation, enables host cell fusion

Human coronaviruses (hCoVs) are enveloped viruses with a positive-sense, single-stranded RNA genome [5]. HCoVs genome size is one of the largest among RNA viruses, ranging from 26.4 to 31.7 kilobases. Viral particles and envelope average diameters are around 125 nm and 85 nm, respectively. On electron microscopy, hCoVs show a characteristic club-shaped spikes that projects from their surface, creating an image reminiscent of the solar corona, from which their name originates [6]. The viral envelope consists of a lipid bilayer, in which the membrane (M), envelope (E) and spike (S) structural proteins are anchored (Fig. 1a) [5–7]. Inside the envelope, viral genome is enclosed, i.e., a ribonucleoprotein (RNP) core, comprising the nucleocapsid protein (N) that acts as a scaffold around the 29,900 nucleotides of RNA. The M and E proteins play a central role in forming the viral envelope and providing the structural integrity [7]. The surface spike (S) belongs to a class I fusion proteins which mediate the receptor binding and the fusion between virus and host cell membranes [8]. The S protein is composed by the S1 subunit, which forms the head of the spike and hosts the receptor-binding domain (RBD), and by the S2 subunit, the stem which anchors the spike to the viral envelope and, following protease activation, enables host cell fusion (Fig. 1b) [8, 9]. After cell entry, viral genome is released into the cell cytoplasm, host ribosomes begin to translate the first reading frame from the viral genome and then via the neo-formed RNA-dependent polymerases, the numerous sub-genomic RNAs are transcribed and then translated [10, 11]. Following genomic RNA replication, the viral structural proteins E and M move along the secretory pathway into the Golgi compartment and maturation of structural proteins occurs. M proteins direct most protein interactions required for assembly of viruses, whilst E proteins are involved in several other aspects of the virus’ life cycle, including envelope formation and budding [7, 11]. In addition to the 4 main structural proteins, hCoVs possess 16 non-structural proteins which assemble to form a multi-protein replicase–transcriptase complex (RTC). RTC promotes viral RNA replication, favors viral survival through inhibition of innate immunity responses, and enhances virulence power [7, 12]. Progeny viruses are released from the host cell by exocytosis through secretory vesicles. In humans, hCoVs infections can affect the respiratory, gastrointestinal, liver and central nervous systems [11, 12]. SARS-CoV and the novel SARS-CoV-2 share 79.5% sequence identity [5, 13–15] and this explains why not only similarities, but also differences can be detected in the epidemiology and clinical features in the disorders they cause [12, 14, 16]. Structural–functional analysis has identified differences in the mechanisms involved in host cell infection which could partially explain the dissimilarity in efficiency and speed of virus transmission between SARS-CoV and SARS-CoV-2.

## SARS-CoV and SARS-CoV-2 host cell infection receptor recognition

**Fig. 2:**
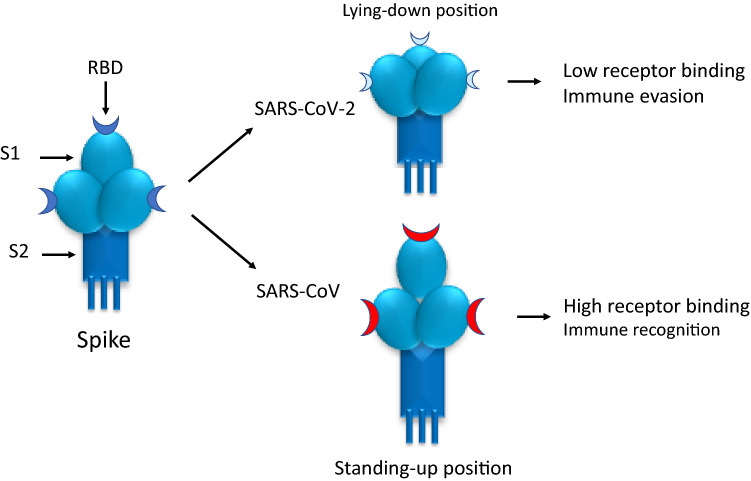
Receptor-binding domain (RBD) of the S protein may constantly switch between a “lying-down” and a “standing-up” position. In SARS-CoV-2, RBD is mostly in the “lying-down” position, a state associated with not only ineffective receptor binding, but also immune evasion. In SARS-CoV, RBD is mostly in “standing-up” position, a state associated with not only high effective receptor binding, but also immune recognition

Receptor recognition is an important determinant of hCoVs infection and pathogenesis. The specific surface protein that provides the entry door in human cells for both SARS-CoV and SARS-CoV-2 is angiotensin-converting enzyme 2 (ACE2) [17–19]. The first difference between the two SARS-CoVs is that SARS-CoV-2 receptor-binding domain (RBD) has a higher ACE2-binding affinity, a characteristic which could lead to a more efficient cell entry [19]. However, ACE2-binding affinity of the entire SARS-CoV-2 S protein seems to be comparable to or even lower than that of SARS-CoV entire S protein. This observation suggests that SARS-CoV-2 RBD, even though more potent, is probably less exposed than SARS-CoV RBD [4]. There have been conflicting reports in the literature on the ACE2-binding affinities of the two SARS-CoVs spike proteins, probably because RBD constantly switches between a “standing-up” position and a “lying-down” position (Fig. 2a) [19, 20]. Evaluation by cryo-electron microscopy (Cryo-EM) of the crystal structure of the two SARS-CoVs RBD, complexed with ACE2 receptors, showed subtle, but functionally important differences [21]. SARS-CoV-2 RBD was mostly in the “lying-down” position, a state associated with ineffective receptor binding [21]. This observation was confirmed by flow cytometry in a different study [22]. In contrast to SARS-CoV-2 spike, Cryo-EM studies showed that in SARS-CoV spike protein, the RBD is mostly in the “standing-up” state [23]. As shown in an animal study, the lower accessibility of the SARS-CoV-2 RBD in “lying-down position”, a state associated with less effective receptor binding, may favor the immune evasion of SARS-CoV-2 as one of the conformational masking strategies. In mice, sera from SARS-CoV-infected animals poorly neutralized SARS-CoV-2 pseudovirus entry into host cells but they bound with high-affinity SARS-CoV RBD and potently neutralized SARS-CoV pseudovirus entry [4, 24, 25]. Conflicting reports on SARS-CoV-2 pathogenicity may also be related to genetic differences in the expression of the SARS-CoV-2 host cell entry factors among individuals and between populations [26, 27]. Indeed, to maintain its high infectivity while keeping its RBD less accessible, SARS-CoV-2 relies on a second strategy, i.e. host protease activation.

## Proteolytic activation

**Fig. 3:**
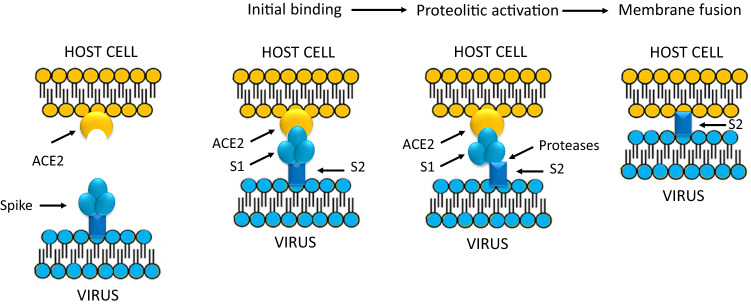
After initial binding of the ACE2 receptor, SARS-CoV spike is proteolytically activated and enzymatically cleaved at the S1/S2 level. S1 than dissociates from S2 and the truncated 2 subunit of the Spike protein facilitates fusion of viral and cellular membranes [28, 38]

After initial receptor binding, hCoVs need to fuse their envelope with the host cell membrane to deliver the viral nucleocapsid into the target cells (Fig. 2b) [28–31]. To fuse membranes, SARS-CoV spike needs to be proteolytically activated at the S1/S2 level. The S2 subunit, cleaved from S1 subunit by host cell proteases, facilitates membrane fusion, bringing the virion into the host cells (Fig. 3) [28]. The major proteases involved in the two SARS-CoVs entry are the cell surface transmembrane protease serine 2 (TMPRSS2) and the lysosomal proteases cathepsins [28, 29, 32]. SARS-CoV S proteins contains cleavage sites for both TMPRSS2 and cathepsins. In experimental SARS-CoV infection, inhibition of both proteases is required to block SARS-CoV entry in cell cultures, however, only TMPRSS2 activity seems to be essential for inhibiting viral replication and spread [29, 32]. An important difference between the two SARS-CoVs is that SARS-CoV-2 S proteins contains also a furin-like cleavage site, absent in the SARS-CoV S protein [4, 29, 32, 33]. Cleavage of S protein by furin at the S1/S2 site is an essential process for cell–cell fusion and SARS-CoV-2 entry into human lung cells [29, 32–34]. Furin pre-activation provides a gain-of-function for a more efficient spreading, enhancing SARS-CoV-2 entry into cells with relatively low expressions of TMPRSS2 and/or lysosomal cathepsins [4, 29, 32–34]. However, protease cleavage of CoVs spikes leads to major structural rearrangement of the S2 subunit [35]. This process, which is irreversible, may reduce entry efficiency in some types of cells with high expressions of TMPRSS2 and cathepsins, as shown in in vitro studies performed in different cell line cultures [36]. Examining the whole native SARS-CoV-2 architecture by transmission electron microscopy, it has been shown that many S molecules had already undergone the structural changes associated with less efficiency [4, 37]. As shown in in vitro experiments, the reduced viral entry capacity induced by furin pre-activation may be more relevant for “not fresh” virus particles [4]. Indeed, conformational modifications of the S molecules, which may slowly occur spontaneously, can be facilitated by a variety of environmental factors, such as physical force, high temperature or chemicals [4]. Like SARS-CoV, also SARS-CoV-2 spread depends on TMPRSS2 activity, but in vitro studies showed that a TMPRSS2 inhibitor, camostat mesylate [38], only partially blocked SARS-2-S-driven entry into human epithelial cell line cells. This finding suggests that that furin-mediated precleavage at the S1/S2 site in infected cells could promote subsequent TMPRSS2-dependent entry into target cells [32]. However, being a clinically proven and commercial serine protease inhibitor, camostat mesylate might be helpful for clinicians at intensive care unit treating severely ill COVID-19 patients [39]. Finally, SARS-CoV-2 also infect endothelial cells and, during COVID-19, one of the serine-proteases activated is thrombin. Part of SARS-CoV-2 pathogenesis is caused by enzymatic activation of the clotting cascade by thrombin activation at the endothelial surface of capillaries with a significant risk of thrombotic complications, ranging from microvascular thrombosis, to venous thromboembolic, disseminated intravascular coagulation and endothelial plasma leakage and thus alveolar obstruction [40]. The growing awareness and mechanistic understanding of the prothrombotic state of COVID-19 patients is driving efforts to more stringent diagnostic screening and to the early institution of antithrombotic drugs for both prevention and treatment of thrombotic complications [40, 41].

## Conclusions

Both SARS-CoV and SARS-CoV-2 use human ACE2 as entry receptor and human proteases as entry activators. In vitro studies have identified strategies that SARS-CoV-2 adopts to infect human cells that potentially contribute to wide spread of the virus and to immune evasion. These cell entry mechanisms may represent substantial target for host immune surveillance and provide insight into possible intervention strategies to fight the pandemic induced by this novel agent.
